# Effects of chest wall compression on expiratory flow rates in patients with chronic obstructive pulmonary disease

**DOI:** 10.1590/bjpt-rbf.2014.0145

**Published:** 2016-03-15

**Authors:** Masafumi Nozoe, Kyoshi Mase, Tomoyuki Ogino, Shigefumi Murakami, Sachie Takashima, Kazuhisa Domen

**Affiliations:** 1 Department of Physical Therapy, Faculty of Nursing and Rehabilitation, Konan Women's University, Morikita-machi, Higashinada-ku, Kobe, Japan; 2 Department of Rehabilitation, Hyogo College of Medicine Sasayama Medical Center, Kurooka-cho, Sasayama-shi, Hyogo, Japan; 3 Department of Physical Medicine and Rehabilitation, Hyogo College of Medicine, Mukogawa-cho, Nishinomiya-shi, Hyogo, Japan

**Keywords:** physical therapy modalities, chronic obstructive pulmonary disease, peak expiratory flow rate

## Abstract

**Background:**

Manual chest wall compression (CWC) during expiration is a technique for removing airway secretions in patients with respiratory disorders. However, there have been no reports about the physiological effects of CWC in patients with chronic obstructive pulmonary disease (COPD).

**Objective:**

To compare the effects of CWC on expiratory flow rates in patients with COPD and asymptomatic controls.

**Method:**

Fourteen subjects were recruited from among patients with COPD who were receiving pulmonary rehabilitation at the University Hospital (COPD group). Fourteen age-matched healthy subjects were also consecutively recruited from the local community (Healthy control group). Airflow and lung volume changes were measured continuously with the subjects lying in supine position during 1 minute of quiet breathing (QB) and during 1 minute of CWC by a physical therapist.

**Results:**

During CWC, both the COPD group and the healthy control group showed significantly higher peak expiratory flow rates (PEFRs) than during QB (mean difference for COPD group 0.14 L/sec, 95% confidence interval (CI) 0.04 to 0.24, p<0.01, mean difference for healthy control group 0.39 L/sec, 95% CI 0.25 to 0.57, p<0.01). In the between-group comparisons, PEFR was significantly higher in the healthy control group than in the COPD group (-0.25 L/sec, 95% CI -0.43 to -0.07, p<0.01). However, the expiratory flow rates at the lung volume at the PEFR during QB and at 50% and 25% of tidal volume during QB increased in the healthy control group (mean difference for healthy control group 0.31 L/sec, 95% CI 0.15 to 0.47, p<0.01: 0.31 L/sec, 95% CI 0.15 to 0.47, p<0.01: 0.27 L/sec, 95% CI 0.13 to 0.41, p<0.01, respectively) but not in the COPD group (0.05 L/sec, 95% CI -0.01 to 0.12: -0.01 L/sec, 95% CI -0.11 to 0.08: 0.02 L/sec, 95% CI -0.05 to 0.90) with the application of CWC.

**Conclusion:**

The effects of chest wall compression on expiratory flow rates was different between COPD patients and asymptomatic controls.

## BULLET POINTS

- We compared the effects of CWC on expiratory flow rates in patients with COPD and asymptomatic controls.
- It was confirmed that PEFR increased during CWC in COPD patients; however, PEFR changes during CWC were lower in COPD patients than in healthy subjects.
- Although CWC appears to be less effective in increasing absolute expiratory flow rates in COPD patients than in healthy subjects, the PEFR, which is more effective for removing secretions, did increase in the COPD group.

## Introduction

Manual chest wall compression (CWC) during expiration is a technique for removing airway secretions^1^^,^ 2. It is known that the main physiological mechanism for removing secretions is increased expiratory flow rates due to increased pleural pressure^3^^-^ 5 and stretching of intercostal muscles by means of manual thoracic compression applied during exhalation^6^. Several studies have also shown that CWC increased expiratory flow rates, improved removal of airway secretions, and improved gas exchange and pulmonary mechanics^2^^,^ 6 ^-^ 8 in patients on mechanical ventilation^6^^-^ 11, as well as in patients with cystic fibrosis^3^, in animal models^8^^,^ 12. Some studies showed positive effects^8^^,^ 13, but another study showed negative effects on clinical outcomes with CWC, such as expiratory flow limitation (EFL)^6^.

It has also been reported that many physical therapists often use manual chest physical therapy techniques in patients with acute exacerbations of chronic obstructive pulmonary disease (COPD)^14^. It is great of importance for physical therapists to know the physiological effects of CWC in patients with COPD. It may also be harder to increase expiratory flow rates with CWC in COPD patients, because many COPD patients often show difficulty increasing expiratory flow rates because of airflow limitation^15^. However, there have been no reports of the physiological effects of CWC in patients with COPD.

The purpose of this study was to compare the effects of CWC on expiratory flow rates in patients with COPD and asymptomatic controls. We hypothesized that expiratory flow rates during CWC are harder to increase in COPD patients.

## Method

### Participants

This study was approved by the Hyogo College of Medicine, Nishinomiya-shi, Hyogo, Japan Ethics Committee (approval number 1189). Written, informed consent was obtained from all eligible participants. The study included fourteen clinically stable patients with stage I to IV COPD according to the Global Initiative for Chronic Obstructive Lung Disease guidelines (COPD group) who were receiving pulmonary rehabilitation at the Hyogo College of Medicine Sasayama Medical Center between October 2012 and September 2013 and who could perform spirometric testing according to the ATS/ERS Task Force Guidelines^16^. Forced expiratory volume in the first second (FEV_1_) and forced vital capacity (FVC) were expressed as predicted percentage values for age, sex, and height, established by the Japanese Respiratory Society^17^. The patients were clinically stable for ≥4 weeks. Exclusion criteria were suspected asthma, other systemic conditions that could contribute to dyspnea or exercise limitation (e.g. heart failure or metabolic disorders), and neuromuscular comorbidity limiting all measurements or non-approval for study participation. All COPD patients continued their regular treatment (all COPD patients used inhaled long-acting beta 2-agonists or long-acting muscarinic antagonists, and one patient used an inhaled corticosteroid). No changes in the medications were made for the purpose of the study. Fourteen age-matched healthy subjects were also consecutively recruited from the local community (healthy control group).

### Measurement procedures

All measurements were collected with the subjects in supine position. Airflow rates and lung volume changes during 1 minute of quiet breathing and during 1 minute of CWC were measured via a mouth filter (PIF-1A; MINATO Medical Science, Osaka, Japan) with a heated pneumotachograph (AE300-s; MINATO Medical Science). A mouth filter was used to avoid cross-infection. CWC was applied to each subject during expiration by a male physical therapist with 8 years of clinical experience in pulmonary physical therapy. The physical therapist stood on the right side of the subject and placed both hands on the subject's upper rib cage (upper part from the sixth rib). CWC was started from the beginning of expiration to the end of expiration. The highest tolerable level of force was applied to the subject's chest wall then released as soon as the subject began inspiration. The subject was asked to avoid actively expiring during the application of CWC but to inspire freely. All subjects also performed the inspiratory capacity (IC) maneuver at the start during quiet breathing (QB) and at the end during CWC to correct the volume measuring errors ("drift")^15^.

Airflow and lung volume data were examined using an analysis system (PowerLab, ADInstruments, Castle Hill, NSW, Australia). The last five breaths during QB and CWC were analyzed, and the mean values for Ti (inspiratory time), Te (expiratory time), Ttot (total breathing cycle time), Ti/Ttot (duty cycle), Vt (tidal volume), PIFR (peak inspiratory flow rate), PEFR (peak expiratory flow rate), PEFR/PIFR, Vt/Ti (mean inspiratory flow rate), and Vt/Te (mean expiratory flow rate) were obtained for each subject.

Flow volume (FV) curve analysis was also performed by calculating the average FV curves from the last five breaths during QB and CWC^18^. The same analysis system (PowerLab, ADInstruments) was also used for averaging FV curves. The expiratory flow rate changes during QB and CWC were then examined at the same lung volume (PEFR during QB and at 50% and 25% of tidal volume during QB) and any overlap in the regions of the FV curves was determined. The presence of overlap was defined as a difference in the airflow rates within 5% between the two FV curves at the same lung volume.

### Sample size calculation

The effects of CWC in COPD patients have not been reported. The sample size was calculated using the differences in PEFR with the application of CWC in the first seven subjects in each group. The mean difference in the between-group comparison was 0.195 L/sec (standard deviation (SD) 0.183). A sample size of 14 subjects per group was thus required for this study to have 80% power with alpha of 0.05.

### Statistical analysis

The results are shown as means±SD. Normality of the measurement data was examined using the Kolmogorov-Smirnov test. The unpaired Student's *t*-test or Mann-Whitney's *U-*test was used to compare demographic characteristics and lung function. The between-group sex distribution was compared using chi-square analysis. Within-group comparisons by CWC intervention were done using the paired Student's *t*-test or Wilcoxon's rank-sum test, and between-group comparisons by CWC intervention were done using the unpaired Student's *t*-test or Mann-Whitney's *U*-test. A chi-square analysis was performed to compare the ratios of subjects who showed overlapping regions between the COPD group and the healthy control group. All tests were performed at a significance level of P<0.05. Analyses were performed with statistical software (SPSS 20, SPSS, Chicago, IL, USA).

## Results

Table 1 shows the baseline characteristics of the study participants. There were significant differences between the groups in pulmonary function.

**Table 1.** Baseline characteristics of the study participants.

	**Healthy control group (n=14)**	**COPD group (n=14)**	**p-value**
Female, n (%)	6 (43%)	5 (36%)	0.70
Age (year)	77 (7)	80 (8)	0.21
Weight (kg)	56 (11)	48 (9)	0.059
Height (cm)	160 (10)	157 (7)	0.41
BMI (kg/m^2)^	22 (3)	19 (3)	0.05
FEV_1_ (L)	2.0 (0.5)	1.1 (0.6)	<0.0001
%FEV_1_ (% predicted)	92 (10)	52 (20)	<0.0001
FEV/FVC (%)	78 (8)	56 (12)	<0.0001
FVC (L)	2.7 (0.6)	1.9 (0.8)	0.007
%FVC (%predicted)	95 (10)	71 (20)	0.0004

BMI: body mass index; FEV_1_: forced expiratory volume on the first second; FVC: forced vital capacity. Continuous data is expressed as mean (SD), categorical data is expressed as number (%).

Table 2 shows the breathing pattern and lung volume changes during QB and CWC. Ti, Te, Ttot, Vt, IC, PIFR, PEFR, and Vt/Ti increased significantly, and Ti/Ttot decreased significantly more during CWC than during QB, both in the COPD group and in the healthy control group, but Vt/Te increased significantly more during CWC than during QB only in the healthy control group. As a result, PIFR/PEFR was not different during QB and CWC in both groups. In the between group comparisons, Vt, PIFR, PEFR, Vt/Ti, and Vt/Te were significantly higher in the healthy control group than in the COPD group.

**Table 2.** Changes in breathing patterns and lung volumes during QB and CWC in both groups.

	**Groups**	**Within-Group differences (95% CI)**	**Between-Group differences (95% CI)**				
	**QB**	**CWC**	**CWC minus QB**	**CWC minus QB**			
COPD (n=14)	Healthy (n=14)	COPD (n=14)	Healthy (n=14)	COPD	Healthy	COPD minus Healthy
Ti (sec)	1.45	1.59	1.70	1.85	0.25*	0.26*	-0.01
	(0.45)	(0.38)	(0.61)	(0.53)	(0.34)	(0.41)	(-0.31 to 0.30)
Te (sec)	2.44	2.37	3.65	3.18	1.21**	0.81**	0.40
	(0.99)	(0.87)	(1.27)	(0.80)	(0.80)	(0.73)	(-0.22 to 1.02)
Ttot (sec)	3.89	3.96	5.35	5.03	1.46**	1.07**	0.39
	(1.37)	(1.20)	(1.74)	(1.17)	(0.94)	(0.99)	(-0.39 to 1.16)
Ti/Ttot	0.38	0.41	0.32	0.37	-0.06**	-0.04*	-0.02
	(0.05)	(0.05)	(0.06)	(0.05)	(0.05)	(0.07)	(-0.07 to 0.03)
Vt (L)	0.61	0.68	0.86	1.22	0.25**	0.55**	-0.30^†^
	(0.22)	(0.17)	(0.29)	(0.46)	(0.17)	(0.39)	(-0.54 to -0.05)
IC (L)	1.54	2.17	1.68	2.39	0.14**	0.22**	-0.08
	(0.71)	(0.58)	(0.77)	(0.55)	(0.14)	(0.16)	(-0.20 to 0.04)
PIFR (L/sec)	0.57	0.56	0.76	0.93	0.19**	0.37**	-0.18^†^
	(0.13)	(0.15)	(0.24)	(0.28)	(0.19)	(0.25)	(-0.36 to -0.01)
PEFR (L/sec)	0.56	0.60	0.70	0.99	0.14**	0.39**	-0.25^‡^
	(0.21)	(0.20)	(0.24)	(0.36)	(0.16)	(0.27)	(-0.43 to -0.07)
PEFR/PIFR	0.97	1.05	0.97	1.06	0	0.01	-0.01
	(0.27)	(0.15)	(0.24)	(0.18)	(0.30)	(0.21)	(-0.22 to 0.19)
Vt/Ti (L/sec)	0.45	0.44	0.52	0.67	0.09**	0.23**	-0.14^‡^
	(0.11)	(0.12)	(0.16)	(0.17)	(0.10)	(0.14)	(-0.23 to -0.04)
Vt/Te (L/sec)	0.26	0.31	0.24	0.40	-0.03	0.09	-0.12^‡^
	(0.08)	(0.10)	(0.06)	(0.15)	(0.06)	(0.12)	(-0.19 to -0.04)

Results expressed as mean (standard deviation) and mean difference and 95% confidence intervals (CI) between measurement conditions. COPD: chronic obstructive pulmonary disease group; Healthy, healthy control group; QB: quiet breathing; CWC: chest wall compression; Ti: inspiratory time; Te: expiratory time; Ttot: total breathing cycle time; Ti/Ttot: duty cycle; Vt: tidal volume; IC: inspiratory capacity; PIFR: peak inspiratory flow rate; PEFR: peak expiratory flow rate; Vt/Ti: mean inspiratory flow rate; Vt/Te: mean expiratory flow rate. *, **: Significant change between QB and CWC (*P*<0.05, *P*<0.01).†, ‡: Significant change between COPD group and healthy control group (*P*<0.05, *P*<0.01).

Table 3 shows the expiratory flow rates at the same lung volume during QB and CWC. The expiratory flow rates at the lung volume at PEFR during QB and at 50% and 25% of tidal volume during QB were higher only in the healthy control group, but not in the COPD group with CWC.

**Table 3.** Changes in expiratory flow rates at the same lung volume during QB and CWC in both groups.

	**Groups**	**Within-Group differences**	**Between-Group differences**				
	**QB**	**CWC**	**CWC minus QB**	**CWC minus QB**			
COPD (n=14)	Healthy (n=14)	COPD (n=14)	Healthy (n=14)	COPD	Healthy	COPD minus Healthy
EFR at PEFR (L/sec)	0.56	0.60	0.62	0.91	0.05	0.31**	-0.26^‡^
	(0.21)	(0.20)	(0.23)	(0.35)	(0.11)	(0.27)	(-0.42 to -0.09)
EFR at 50% Vt (L/sec)	0.44	0.53	0.43	0.84	-0.01	0.31**	-0.32^‡^
	(0.20)	(0.19)	(0.19)	(0.34)	(0.16)	(0.27)	(-0.50 to -0.15)
EFR at 25% Vt (L/sec)	0.28	0.40	0.29	0.67	0.02	0.27**	-0.25^‡^
	(0.09)	(0.15)	(0.17)	(0.30)	(0.12)	(0.34)	(-0.40 to -0.10)

Results expressed as mean (standard deviation), and mean difference and 95% confidence intervals (CI) between measurement conditions. COPD: chronic obstructive pulmonary disease group; Healthy: healthy control group; QB: quiet breathing; CWC: chest wall compression; EFR: expiratory flow rates; PEFR: peak expiratory flow rates; Vt: tidal volume. **: Significant change between QB and CWC (*P*<0.01). ‡: Significant change between COPD group and healthy control group (*P*<0.01).

**Figure 1.**
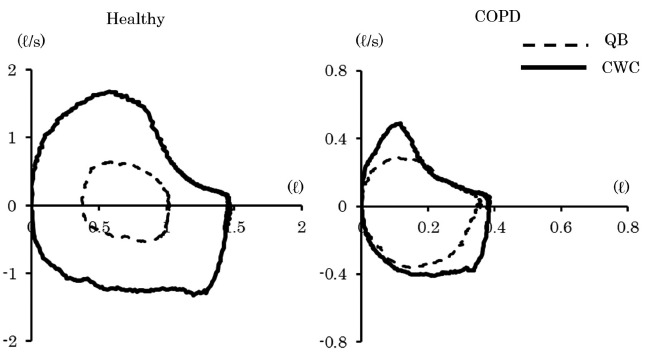
Representative flow-volume curves during QB and CWC. The left side shows a healthy subject, and the right side shows a COPD patient. QB: quiet breathing; CWC: chest wall compression; COPD: chronic obstructive pulmonary disease.

Figure 1 shows representative FV curves during QB and CWC in the COPD group and the healthy control group. The left FV curves in the healthy control group show increased expiratory flow rates at all lung volumes. In contrast, the right FV curves in the COPD group show increased expiratory flow rates during CWC at the beginning of expiration, but not at the end of expiration, and the FV curves had overlapping regions. Less than half of the subjects in the healthy control group (6/14 subjects; 43%) had overlapping regions in the FV curves, but almost all subjects in the COPD group had overlapping regions (13/14 subjects; 93%, P<0.05). All overlapping regions were seen at the end of expiration.

## Discussion

In this study, the effects of CWC were examined in COPD patients, and it was confirmed that PEFR increased during CWC in these patients. However, PEFR changes during CWC were lower in COPD patients than in healthy subjects. The reason for the difference may have been the presence of expiratory flow limitation (EFL) in the COPD patients. With EFL, the expiratory flow rates never increase with increased pleural pressure, and this is often seen in patients with severe COPD^19^. Ninane et al.^20^ reported assessment of EFL by studying whether the expiratory flow rates could be increased by increased pleural and abdominal pressures with abdominal compression during expiration. In the present study, CWC also increased pleural pressure by upper rib cage compression, but the participants whose expiratory flow rates did not increase during CWC probably had EFL, though the area of compression differed from that of Ninane et al.^20^. In fact, almost all COPD patients showed overlapping regions in the FV curves during QB and CWC, which suggests that it is difficult for COPD patients to increase expiratory flow rates during CWC. One more reason to consider for the discrepancy in PEFR changes between healthy and COPD participants is the increase in Vt with CWC. Generally, increasing Vt provides higher elastic forces at the start of exhalation, therefore healthy subjects have a tendency to increase expiratory flow rates more than COPD patients do.

In this study, expiratory flow rate changes during CWC were also examined using FV curves. PEFR and Vt have often been measured during various chest physical therapy techniques^2^^,^ 4 ^,^ 7, but FV curves during these interventions have not been previously studied, except in a recent study on patients on mechanical ventilation^6^. The advantage of using FV curve assessment is to examine the expiratory flow changes at absolute volume in peripheral airway regions^3^. The expiratory flow rates at the lung volume at PEFR during QB and at 50% and 25% of tidal volume during QB increased only in the healthy control group, but not in the COPD group. Moreover, almost all COPD patients showed overlapping regions in the FV curves. This showed that it is difficult for COPD patients to increase expiratory flow in peripheral airways with CWC.

The present results showed that PEFR/PIFR did not change with CWC in the COPD group or in the healthy control group. McCarren and Alison^3^ reported that PEFR/PIFR increased during vibration to 1.51 in CF patients. It was concluded that vibration was useful for CF patients because the PEFR/PIFR value needs to increase to more than 1.1 to remove secretions^3^. However, there were some differences between their methods and the present methods. They asked their subjects to inspire to total lung capacity as slowly as possible^3^. On the other hand, they did not ask subjects to inspire slowly in their other study of normal healthy subjects^5^. In the present study, the participants were instructed to inspire freely, therefore it was not possible to determine inspiratory lung volume and breathing speed. McCarren et al.^5^ reported that not only PEFR but also PIFR increased during CWC in normal subjects, so that PEFR/PIFR decreased more during CWC (to 0.64) than during QB (to 0.72). These results suggest that CWC must be combined with slow deep inspiration for effective removal of secretions. In the present results, Ti, Te, and Ttot also increased during CWC in both groups. Such changes were also seen during pursed-lips breathing (PLB) in COPD patients^21^^,^ 22 because of the increased inspiratory effort during PLB. We hypothesized that increased inspiratory effort was the result of maintaining ventilation, because decreased respiratory frequency was seen not only during PLB but also CWC. Furthermore, we believe that the reason for the greater PIFR and Vt/Ti changes with CWC in the healthy control group compared to the COPD group was the presence of hyperinflation in COPD patients. It is known that lung hyperinflation leads to decreased inspiratory flow reserve by the increased elastic recoil pressure of the lung or the decreased inspiratory muscle-generated force^15^.

In the present results, tidal volume was increased with CWC in both groups, but the increase was greater in the healthy control group than in the COPD group. We thought this difference was also explained by the differences in Vt/Ti between the groups. In contrast, IC increased with CWC in both groups. It is known that IC changes are associated with changes in end expiratory lung volume, so this technique may reduce hyperinflation, such as that shown in PLB^21^^,^ 22. In fact, CWC has been used as a technique to reduce dyspnea by reducing hyperinflation, particularly in Japanese clinical practice^23^.

### Limitations of the study

CWC effects were measured in the supine position, but this position may affect the expiratory flow changes. Koulouris et al.^24^ reported that COPD patients often have EFL, particularly in the supine position. The decreased functional residual capacity (FRC) in the supine position decreases elastic lung recoil and expiratory flow reserve. On the other hand, McCarren et al.^4^ applied CWC to their subjects in the lateral recumbent position. FRC values are higher in the lateral recumbent position than in the supine position^25^, so these differences may have affected the present results. However, the supine position was chosen because it provides more powerful chest compression, as shown by Toussaint et al.^26^. Moreover, CWC was applied to the upper rib cage in the present study, even though it was applied to the lower rib cage in other studies^2^^-^ 5. It is more difficult to apply CWC to the lower rib cage due to the presence of breasts in women; since there were subjects of both sexes in this sample, applying CWC to the upper rib cage was an alternative to achieve better standardization. Some of the healthy subjects in the present study also had overlapping regions in their FV curves. This may be due to age-related changes. The present subjects included many elderly persons, and their mean age was also very advanced. Since it is known that elastic lung recoil pressure and FRC decrease with age^27^, some of the present healthy subjects may have had EFL. However, expiratory flow rates and tidal volume were different between the COPD group and the healthy control group. Thus, there appears to be a difference in the effects of CWC depending on the presence of COPD, even if age-related effects are excluded.

Another possible limitation was that changes in the amounts of secretions with CWC intervention and the longitudinal effects of CWC were not measured. Since the subjects were not asked to inspire to total lung capacity, the lung volume effects could not be controlled. It has been reported that chest physical therapy technique has low reliability^28^. In the present study, the effects of CWC performed by one physical therapist were examined, so that the same results may not be generalized to other physical therapists. Moreover, either the physical therapist or the assessor were not blinded. This lack of blinding may also affect the results of the study.

### Clinical implications

In this study, peak expiratory flow rates, but not mean expiratory flow rates, increased in COPD patients. Volpe et al.^29^ reported that peak expiratory and inspiratory flows are the key factors in secretion removal, not mean expiratory and inspiratory flows. Therefore, we believe that it would be effective to use CWC in patients with COPD to remove secretions when combined with slow inspiration, even though it would be less effective than in healthy subjects. We also showed that IC increased with CWC, so this technique may reduce hyperinflation and dyspnea.

## Conclusion

The present results showed higher PEFR during CWC than during QB, both in COPD patients and in healthy subjects, but absolute expiratory flow rates during CWC increased only in healthy subjects, not in COPD patients. As a result, PEFR and Vt/Te increased with CWC more in healthy subjects than in COPD patients. Although CWC appears to be less effective in increasing absolute expiratory flow rates in COPD patients than in healthy subjects, the PEFR, which is more effective for removing secretions, did increase in the COPD group.
